# Paediatric poisoning fatalities: toxicological, forensic, and child protection considerations

**DOI:** 10.3389/ftox.2026.1987557

**Published:** 2026-10-13

**Authors:** Emina Dervišević, Temima Ajanović, Amina Zorlak-Čavčić, Giuseppe Davide Albano, Claudia Trignano, Anita Galić Mihić, Almir Fajkić, Ferid Krupić, Stefano D’Errico

**Affiliations:** 1 Deparment of Forensic Medicine, Faculty of Medicine, University of Sarajevo, Sarajevo, Bosnia and Herzegovina; 2 Department of Chemistry, Faculty of Science, University of Sarajevo, Sarajevo, Bosnia and Herzegovina; 3 Section of Legal Medicine, Department of Health Promotion, Mother and Child Care, Internal Medicine and Medical Specialties, University of Palermo, Palermo, Italy; 4 Department of Biomedical Sciences, University of Sassari, Sassari, Italy; 5 Institut of Forensic Medicine and Criminalistics University of Zagreb, Zagreb, Croatia; 6 Department of Pathophysiology, Faculty of Medicine, University of Sarajevo, Sarajevo, Bosnia and Herzegovina; 7 Departments of Orthopaedics, Institute of Clinical Sciences, Sahlgrenska Academy, University of Gothenburg, Gothenburg, Sweden; 8 Department of Anaesthesiology, Institute of Clinical Sciences, Sahlgrenska Academy, University of Gothenburg, Gothenburg, Sweden; 9 Department of Medical, Surgical and Health Sciences, University of Trieste, Trieste, Italy

**Keywords:** abuse, mortality, multidisciplinary approaches, neglect, paediatric poisoning

## Abstract

Childhood poisoning remains a significant global public health concern and continues to rank among the leading causes of unintentional injury-related deaths in children aged 0–14 years. Even though many of these exposures are thought to be accidental, it can occasionally be challenging to determine the reason and purpose behind a particular occurrence. This review examines the epidemiology, mechanisms, and classifications of paediatric toxicological deaths, with emphasis on accidental, abuse-neglect-related -associated poisonings in children. The review discusses global and regional trends in paediatric poisoning mortality, highlighting age-specific risk patterns and the most commonly implicated toxic agents. It further explores the medico-legal classification of toxicological deaths, differentiating between natural and unnatural causes and emphasizing the critical role of forensic toxicology in determining the manner of death. Particular attention is given to intentional poisoning associated with child abuse, including drug-facilitated abuse and Munchausen syndrome by proxy, alongside key clinical and forensic indicators. The review also addresses toxic exposures resulting from neglect, including environmental toxins such as lead and carbon monoxide, unsafe living conditions, and patterns of chronic *versus* acute exposure. In addition, accidental poisonings involving household chemicals and medications are examined, with discussion of contributory factors such as inadequate supervision and unsafe packaging. Prevention strategies, public health interventions, and the importance of early recognition are emphasized throughout. This review highlights the need for multidisciplinary approaches involving healthcare professionals, forensic specialists, and public health systems to reduce paediatric toxicological mortality and improve child safety outcomes.

## 1 Introduction

Pediatric forensic toxicology plays a fundamental role in investigating suspected poisoning-related illnesses and deaths by identifying and interpreting toxic substances in biological specimens from infants and children (Franzin et al., 2024). Interpretation of toxicological findings requires consideration of developmental physiology, age-related pharmacokinetics, and metabolic differences that distinguish children from adults. Accurate interpretation of toxicological findings also requires integration with the clinical history, scene investigation, and autopsy findings to distinguish accidental poisoning from neglect, abuse, or intentional poisoning (Bharti et al., 2024).

Childhood poisoning remains a significant global public health concern and continues to rank among the leading causes of unintentional injury-related deaths in children aged 0–14 years. Although most pediatric poisonings are considered accidental, determining the exact circumstances and intent may be challenging in individual cases. Homicidal poisoning is an uncommon cause of poisoning in children, although the true incidence remains uncertain because of the limited available evidence. In cases of unexplained or suspicious pediatric deaths, medicolegal investigations integrate scene findings, clinical history, autopsy examination, and toxicological analyses to establish the cause and manner of death. In this context, pediatric forensic toxicology provides objective scientific evidence to determine whether toxic exposure caused or contributed to death and to distinguish accidental poisoning from neglect or intentional harm (Aquila et al., 2023). Pediatric toxicological investigations differ from those in adults because developmental physiology, immature metabolic pathways, and age-dependent pharmacokinetics influence the absorption, distribution, metabolism, and elimination of toxic substances. Interpretation is further complicated by limited biological sample volumes, diverse exposure scenarios, and difficulties in relating toxicological concentrations to toxicity thresholds. Unexpected and unexplained pediatric deaths remain an important global healthcare concern (Goldstein et al., 2023). Despite the limitations of post-mortem investigations, forensic pathologists play a crucial role in establishing the cause and manner of death. Therefore, a multidisciplinary approach integrating forensic pathology and toxicology is essential in the investigation of pediatric poisoning deaths.

The aim of this review is to provide a comprehensive overview of paediatric toxicological deaths by examining epidemiological patterns, developmental susceptibility, forensic classification, abuse- and neglect-related poisoning, common toxic agents, and prevention strategies from clinical, toxicological, and medico-legal perspectives.

## 2 Literature search strategy

A thorough literature search conducted in PubMed, Scopus, Web of Science, and Google Scholar served as the foundation for this narrative review. Clinical guidelines, systematic reviews, meta-analyses, peer-reviewed papers, and forensic reports that were mostly written in English were taken into consideration. Publications on pediatric poisoning deaths, forensic toxicology, child abuse and neglect, and post-mortem toxicological studies were the main subjects of the review, which took place between January and May 2026. To give a broad picture of current understanding, evidence from many geographical regions was included, including Europe, North America, Asia, and other places where accessible. Clinical practice guidelines, systematic reviews, meta-analyses, narrative reviews, observational studies, case series, case reports, peer-reviewed original research, and relevant forensic reports were considered when they provided information directly applicable to the objectives of the review.

Publications were considered eligible when they addressed children, infants, or adolescents and provided clinically, toxicologically, epidemiologically, or forensically relevant information on poisoning or fatal toxic exposure. Studies concerning adult populations alone, publications unrelated to poisoning or toxic exposure, and papers that did not provide information relevant to pediatric or forensic assessment were excluded. Where mixed-age populations were studied, publications were considered when pediatric findings could be separately identified or were directly applicable to the review question.

The review primarily considered English-language literature, while relevant publications in other languages were considered where accessible and where sufficient information could be obtained for assessment. Both recent and seminal publications were included in order to provide an appropriate balance between current evidence and important foundational studies. Particular emphasis was placed on literature published in recent years to reflect contemporary patterns of pediatric poisoning, advances in analytical toxicology, and current approaches to forensic investigation.

## 3 Epidemiology of paediatric toxicological deaths

Several factors influence the etiology of poisoning in children, such as socio-economic status, geographic distribution, cultural practices, family dynamics and climate conditions (Solmaz et al., 2026). There are notable differences in pediatric toxicological mortality patterns between high-income and low- and middle-income countries (Wang et al., 2025). These differences are influenced by variations in toxic substance availability, healthcare access, and reporting and certification practices, which should be considered when interpreting epidemiological data. Reported mortality in pediatric poisoning varies widely across studies, with case-fatality rates ranging from approximately 1.8%–13.6%, and individual hospital-based studies reporting mortality around 3% (e.g., 3.04%) in severe poisoning cases (Dai et al., 2022). Unintentional poisonings are more common in younger children, whereas intentional exposures are more frequently observed in adolescents. The highest risk age group is typically children under 6 years, particularly those aged 2–3 years, due to exploratory behaviour and increased exposure to household toxins (Wojciechowki et al., 2024). In low- and middle-income countries, pesticides are more commonly implicated in fatal cases, while in high-income countries pharmaceuticals represent the leading agents involved in pediatric poisonings. Fatal outcomes in pediatric poisoning are most frequently attributed to organophosphate-induced cholinergic crisis leading to respiratory failure and cardiovascular collapse, central nervous system depression following drug overdose, and corrosive injury causing airway obstruction and hypoxic arrest. These differences highlight the importance of context-specific prevention strategies and forensic approaches in pediatric toxicology (Ahmed et al., 2022).

As seen below, in Figure 1 with a focus on medicines as the primary cause of childhood poisoning, the picture provides a workflow-oriented summary of pediatric toxicological exposure. It provides an overview of pediatric toxicology’s main exposure sources, toxicokinetic processes, clinical results, epidemiological risk factors, laboratory testing, and preventative measures. An integrated workflow summarizing the major determinants, clinical outcomes, laboratory assessment, and prevention strategies in pediatric poisoning is provided in Appendix Supplementary Appendix Figure SA.

**FIGURE 1:**
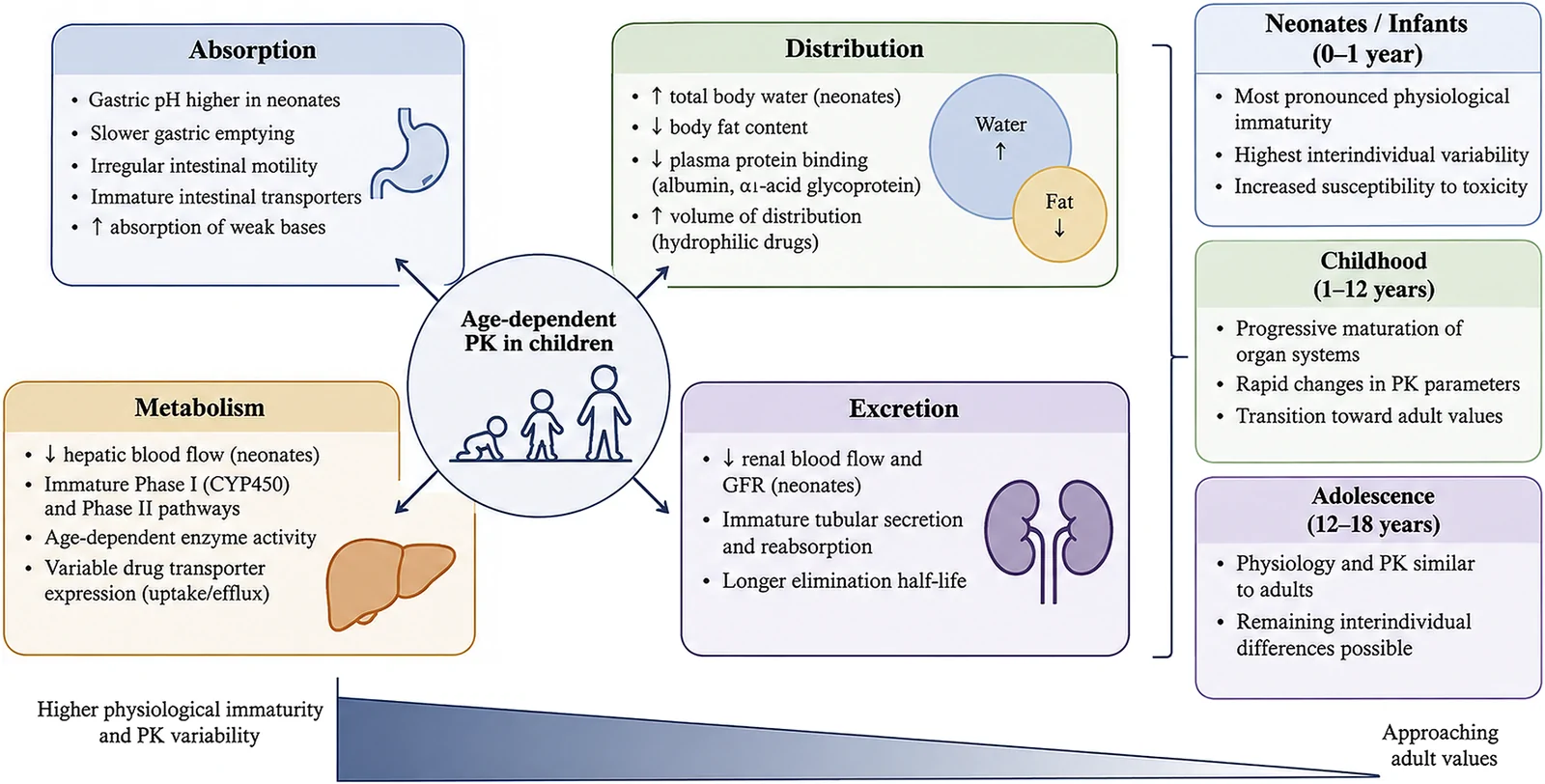
Age-Dependent Pharmacokinetic Changes in Children: Developmental Differences in Absorption, Distribution, Metabolism, and Excretion. Created in BioRender. Ajanovic, T. (2026) https://BioRender.com/23p1gco licensed under Academic license.

### 3.1 Global and regional trends

Global and regional trends in pediatric toxicological deaths show marked differences between high-income and low- and middle-income countries. These differences are influenced by variations in toxic substance availability, healthcare access, and reporting and death certification systems, which should be considered when interpreting epidemiological data (Mottla et al., 2023; Bansal et al., 2024). Reported pediatric poisoning mortality varies widely across regions, with case-fatality rates ranging from less than 2% to more than 10% in some low-resource settings, reflecting disparities in healthcare access and severity at presentation (Gul et al., 2025). Unintentional poisonings predominate in younger children worldwide, whereas intentional exposures, including self-harm and substance misuse, are more frequently reported in adolescents. In low- and middle-income countries, highly toxic agents such as pesticides are more commonly involved in fatal cases, while in high-income countries pharmaceuticals are the predominant substances implicated in pediatric poisonings (Lowry et al., 2017). Although overall mortality trends in high-income settings show gradual decline, likely due to improved regulation, safer packaging, and better clinical management, pediatric toxicological deaths remain a relevant global public health concern. These regional differences underline the importance of context-specific prevention strategies and forensic approaches in pediatric toxicology (Bansal et al., 2024).

However, as variations in reporting methods, the availability of toxicological testing, and death certification procedures may significantly affect the reported fatality rates, direct comparisons between studies should be viewed with caution (Shapiro-Mendoza et al., 2017).

### 3.2 Age-specific risk patterns

Age-specific trends in pediatric toxicological mortality show distinct variations in the toxic substances, exposure conditions, and intent. Poisoning incidents in infants and toddlers (0–5 years old) are mostly unintentional and take place in home settings. They frequently result from exploratory behavior and unintended consumption of drugs, household chemicals, or environmental contaminants. Due to physiological immaturity, which includes underdeveloped metabolic pathways and decreased body mass, this age group is more susceptible to harmful effects, which can lead to rapid clinical deterioration and fatal outcomes even after relatively low-dose exposures (Bansal et al., 2024). Although the incidence of lethal poisoning is often lower in school-aged children (6–12 years), exposures can still happen due to environmental factors, incorrect medicine usage, or unintentional ingestion. Increased independence and behavioral curiosity raise the risk, but supervision usually acts as a buffer (Wojciechowki et al., 2024). However, severe cases in this age group may still progress to respiratory depression or multisystem organ dysfunction depending on the toxic agent and delay in treatment. Adolescents (13–18 years old), on the other hand, show a clear epidemiological shift, with toxicological deaths more often linked to purposeful exposures, such as substance abuse and self-poisoning. Alcohol, illegal substances, and pharmaceuticals—especially those that act centrally—are frequently included in this category. These patterns are significantly shaped by psychosocial factors, including risk-taking behavior, peer pressure, and mental health issues (Bansal et al., 2024). In this age group, fatal outcomes are often associated with intentional overdoses leading to central nervous system depression, respiratory failure, or cardiac arrhythmias (Bansal et al., 2024; Qu et al., 2025). The severity of poisoning in young children depends on several factors, including the specific toxic agent, dose or amount of exposure, route of exposure, and individual characteristics (Bentur et al., 2010). These age-related variations emphasize the need for customized preventive measures and age-specific forensic interpretation in pediatric toxicology, particularly when differentiating between intentional and unintentional exposures in death investigations, as shown in Figure 2.

**FIGURE 2:**
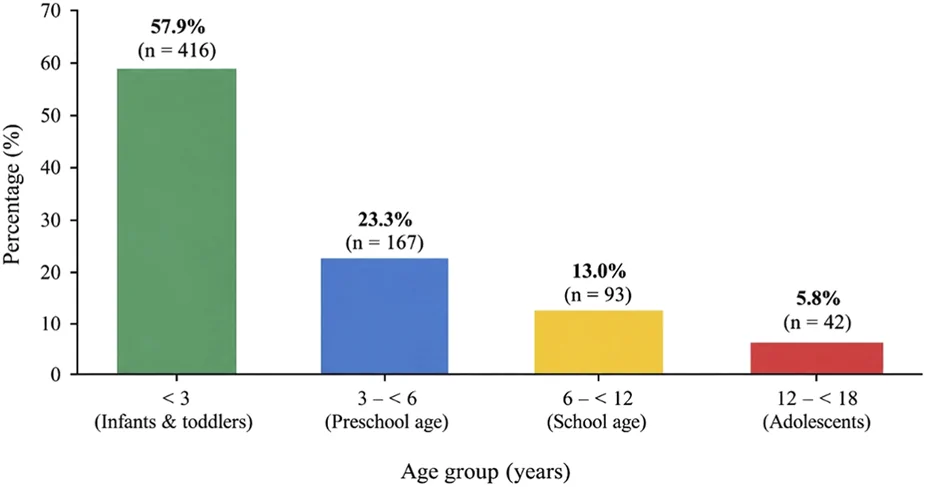
Distribution of pediatric toxic exposures by age group. Data are adapted from Qu et al. (2025), who analyzed 718 pediatric toxic exposure cases and reported that most exposures occurred in children <3 years of age (57.9%), followed by children aged 3–<6 years (23.3%), 6–<12 years (13.0%), and 12–<18 years (5.8%).

### 3.3 Common toxic agents in children

Figure 3 shows the forensic workflow for the investigation of pediatric poisoning fatalities. The range of harmful substances that cause pediatric poisoning vary depending on the child’s age, surroundings, and geographic location. In younger children, the high accessibility of substances within the home explains why pharmaceuticals, household chemicals, and cosmetics are most frequently implicated. Due to incorrect dosage or storage, medications such as sedatives, analgesics, and antipyretics are frequently involved. Hydrocarbons, disinfectants, and household cleaning supplies are important sources of unintentional exposure for this age range (Batchelor and Marriott, 2015). Adolescents, on the other hand, are more likely to be exposed to alcohol, illegal substances, and medications in the context of purposeful poisoning. In this demographic, psychotropic drugs such as benzodiazepines and antidepressants are especially linked to serious or deadly consequences. Clinical management and forensic interpretation both depend on an understanding of the distribution of prevalent hazardous substances, especially when it comes to identifying exposure sources and figuring out how someone died (Dai et al., 2022). Antimicrobials, bisphenols, flame retardants, formaldehyde, heavy metals, PFAS (per- and polyfluoroalkyl substances), phthalates, solvents, and vinyl chloride are among the main groups of common hazardous agents that are related to exposure in children. Although many of these exposures are low-dose and non-fatal, certain heavy metals and industrial chemicals may contribute to severe systemic toxicity and fatal outcomes in cases of significant or chronic exposure (Wojciechowki et al., 2024; De Vries, 2025).

**FIGURE 3:**
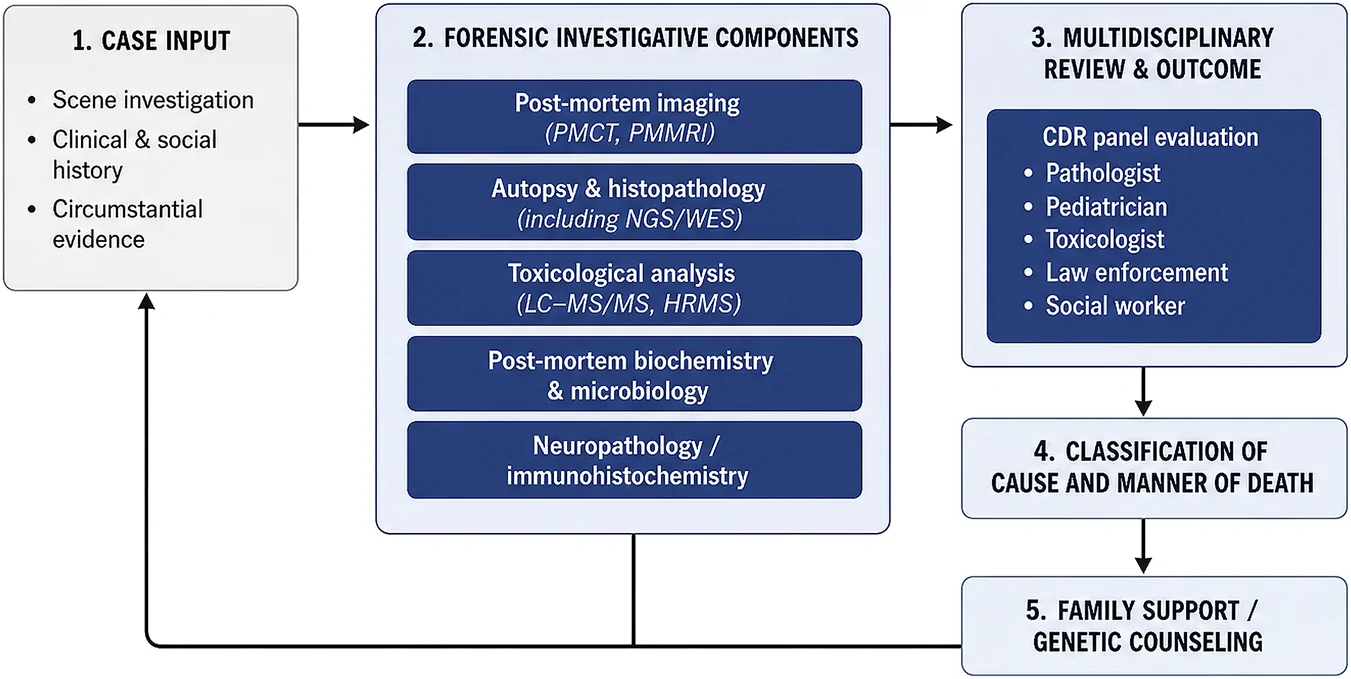
Integrated multidisciplinary framework for the forensic investigation of pediatric poisoning fatalities. The framework integrates scene investigation and case history with post-mortem imaging, autopsy and histopathology, toxicological analysis, post-mortem biochemistry and microbiology, and neuropathological assessment, followed by multidisciplinary case review and determination of the cause and manner of death. Adapted and modified from Chatzinikolaou et al. (2025) under the Creative Commons Attribution (CC BY) license.

## 4 Physiological and pharmacokinetic considerations in children

In order to help formulation development and obtain plasma profile concentrations to support dosing recommendations, pediatric pharmacokinetic clinical studies are typically conducted. Although there is still a significant gap between the amount of clinical trial research and the pediatric burden of disease, recent rules have boosted the number of clinical trials carried out in children during the past 10 years. Even though pediatric patients are now acknowledged as a unique population for medication therapy, childhood brings about a number of physiological changes that may affect a compound’s pharmacokinetics and dynamics. For that reason kids can be separated into numerous classes of age where each group should be viewed as a special population (Dai et al., 2022; Azemi et al., 2012).

Developmental physiology significantly influences toxicity in children, as age-related differences in absorption, distribution, metabolism, and excretion affect both the clinical severity of poisoning and the interpretation of toxicological findings (Qu et al., 2025). These pharmacokinetic differences make pediatric populations particularly vulnerable and require careful consideration in both clinical and forensic toxicology, especially when determining toxic dose-response relationships and cause of death (Bansal et al., 2024; Garg et al., 2025). The principal age-dependent physiological changes affecting the absorption, distribution, metabolism, and excretion of xenobiotics in children are presented in Figure 1, highlighting the progressive transition from pronounced pharmacokinetic variability in infancy toward values approaching those observed in adults during adolescence.

Tables 1–3 summarize the main physiological and pharmacokinetic considerations, medicolegal circumstances, toxic agents, clinical findings, and forensic aspects relevant to pediatric poisoning.

**TABLE 1:** Physiological and pharmacokinetic considerations in children: Chemical, pharmaceutical, and forensic toxicological perspectives.

Physiological/PK factor	Description in children	Chemical aspect	Pharmaceutical implications	Forensic toxicology relevance	References
Absorption	Variable gastric pH, slower gastric emptying, immature skin	Changes in ionization and solubility (pH-dependent stability)	Unpredictable or increased drug absorption	Difficulty in estimating time of exposure and expected concentrations	(Vilaca et al., 2020; Stillhart et al., 2020)
Distribution	Higher total body water, lower plasma protein levels	Altered protein binding and volume of distribution	Increased free drug fraction means higher effect/toxicity	Higher free drug levels and challenges in concentration interpretation	(Ngcobo, 2025; Celestin et al., 2021)
Metabolism	Immature hepatic enzymes (e.g., cytochrome P450)	Slower or variable biotransformation (Phase I/II reactions)	Variable clearance, need for dose adjustment	Prolonged presence of toxins or unexpected metabolites	(Hines, 2016; Zhao et al., 2021)
Excretion	Immature renal function (GFR, tubular secretion)	Reduced elimination of polar compounds	Prolonged drug half-life	Accumulation of substances and increased risk of toxicity	(Blanco et al., 2019; Liabeuf et al., 2025; Rodieux et al., 2015)
Body Composition	Higher percentage of body water, lower fat (especially in neonates)	Altered distribution of hydrophilic/lipophilic substances	Changes in volume of distribution, dosing impact	Different tissue distribution of toxic agents	(Zhang et al., 2023; Hernandez- Gago et al., 2025; Gouju et al., 2023)
Enzyme Ontogeny	Dynamic changes in enzyme activity with age	Variations in enzyme expression and metabolite formation	Altered therapeutic efficacy	Age-dependent variability complicates interpretation	(Wauthier et al., 2007; Subash et al., 2024; Bell et al., 2022)
Blood–Brain Barrier	Underdeveloped in neonates	Easier penetration of lipophilic and small molecules	Increased CNS drug effects	Greater susceptibility to neurotoxic substances	(Niazi, 2023; Wu et al., 2023; Manjari., 2023)
Receptor Sensitivity	Differences in receptor	Altered drug–receptor interactions	Unpredictable therapeutic response	Unexpected toxic effects at lower doses	(Huang et al., 2025; Peris, 2019)

**TABLE 2:** Medicolegal classification of pediatric poisoning fatalities: Circumstances, toxic agents, and key forensic indicators.

Manner of death	Typical cause of death (toxicological mechanism)	Typical circumstances	Common toxic agents	Key forensic indicators	References
Accident	Acute toxic poisoning following unintentional exposure	Exploratory ingestion, unsafe storage of toxic substances, medication errors at home	Household cleaning agents, hydrocarbons, paracetamol, pesticides	Open containers, accessible toxic substances, absence of suicidal intent, consistent caregiver history	(Heard-Garris et al., 2012)
	Intentional self-poisoning	Deliberate overdose in adolescents	Antidepressants, benzodiazepines, opioids, alcohol, illicit drugs	Suicide note, psychiatric history, internet searches, high toxic concentrations	(Pacini et al., 2023; Dutta et al., 2025)
Homicide	Intentional administration of toxic substances by another person	Child abuse, caregiver poisoning, drug-facilitated abuse, Munchausen syndrome by proxy	Sedatives, opioids, insulin, anticoagulants, toxic chemicals	Inconsistent caregiver statements, recurrent unexplained illness, delayed medical care, unexpected toxicological findings	(Fusaroli et al., 2023; Gaither et al., 2024)
Undetermined	Poisoning where available evidence is insufficient to establish the manner of death	Incomplete investigation, advanced decomposition, conflicting findings	Variable	Conflicting autopsy, scene investigation and toxicology results; inability to distinguish accidental from intentional poisoning	(Tieder et al., 2016; Dirbeba., 2018)

**TABLE 3:** Major toxic agents in pediatric poisoning: Exposure patterns, clinical findings, and forensic/medicolegal relevance.

Toxic agent	Exposure pattern	Key mechanism	Acute findings	Chronic findings	Forensic/medicolegal relevance	References
Carbon monoxide (CO)	Acute	Carboxyhemoglobin formation → systemic hypoxia	Rapid collapse, neuro/respiratory failure	Rare, possible neurocognitive sequelae	Unsafe heating/ventilation, preventable domestic hazard	(Rastogi et al., 2019; Schneider, 2023; Mayans, 2019)
Lead (Pb)	Chronic	Accumulation → disruption of synaptic, hematological, metabolic processes	Usually absent or subtle early symptoms	Persistent neurodevelopmental deficits, cognitive/behavioural impairment	Indicator of long-term housing/environmental deterioration	(Tuakuila et al., 2013; Schneider,2023; Mayans, 2019)
Arsenic (As)	Acute or subacute	Inhibition of celullar respiration and enzyme systems → multi-organ toxicity	Severe GI symptoms, shock, arrhythmias, rapid multi-organ failure	Neuropathy, skin changes, organ dysfunction	Environmental contamination or unsafe exposure source	(Karrai et al., 2012; Manzar et al., 2010)

### 4.1 Developmental differences in drug metabolism

Drug metabolism in children is highly age-dependent due to the ongoing maturation of hepatic enzyme systems, involving both phase I and phase II pathways, which together influence drug clearance and toxicity. These developmental differences in phase I and phase II metabolic pathways significantly influence drug clearance, half-life, and toxicity, making age-specific dosing and careful interpretation of toxicological findings essential in pediatric forensic investigations (Johnson, 2005).

### 4.2 Dose–response variability

Dose–response relationships in children vary significantly due to developmental differences in pharmacokinetics and pharmacodynamics. Age-related variability in drug absorption, metabolism, and receptor sensitivity may result in reduced therapeutic effects or increased susceptibility to adverse reactions at standard doses. This variability, together with genetic and physiological factors, contributes to challenges in establishing uniform dosing regimens and highlights the importance of individualized dosing and careful interpretation of toxicological findings in pediatric populations (Johnson, 2005; Hauptman et al., 2017; Hannibal et al., 2025).

### 4.3 Increased vulnerability to toxic substances

Children are particularly vulnerable to toxic substances due to ongoing physiological and developmental maturation. Age-related differences in metabolism, renal excretion, and body composition can increase systemic exposure and prolong toxic effects, even at lower doses compared to adults. In addition, the developing central nervous system is more sensitive to toxic insults, increasing the risk of both acute and long-term adverse outcomes. In severe poisoning cases, these physiological vulnerabilities can facilitate rapid clinical deterioration and progression to fatal outcomes, particularly when exposure involves highly toxic agents or when medical intervention is delayed (Etzel, 2020). These factors highlight the importance of age-specific risk assessment in pediatric toxicology and forensic interpretation of poisoning cases (Flanagan et al., 2005). Table 1 summarizes the main physiological and pharmacokinetic traits of children and their consequences from the viewpoints of chemical, pharmacological, and forensic toxicology.

### 4.4 Pathophysiological basis of fatal decompensation in pediatric poisoning

Fatal outcomes in pediatric poisoning rarely result from the toxic agent alone but from the interaction between the toxin and the limited physiological reserve of the developing organism. Because of reduced physiological and metabolic reserve, toxic exposure in children may rapidly progress to multi-organ dysfunction and systemic decompensation. Respiratory depression, impaired oxygen delivery, and central nervous system suppression represent common final pathways in many fatal poisoning cases, often leading to hypoxia and cardiac arrest regardless of the primary toxic insult. In addition, hepatic and renal dysfunction may further exacerbate toxicity by impairing detoxification and excretion processes, contributing to metabolic instability and organ failure. From a forensic perspective, interpretation of fatal pediatric poisoning requires integration of toxicological findings with autopsy results, clinical history, and scene investigation, as toxic concentrations alone are not sufficient to determine causality or manner of death (Cicco et al., 2020; Grandjean et al., 2014; Alcorn et al., 2002).

## 5 Categories of paediatric toxicological deaths

Depending on the circumstances and purpose of exposure, pediatric toxicological fatalities can be roughly divided into a number of categories. The most prevalent type, especially in younger children, is accidental poisoning, which frequently results from inadvertent absorption of home chemicals, drugs, or environmental contaminants. Including instances of drug-related or poisonous substance-related suicide or self-harm (Filler et al., 2021). An accidental poisoning event should not, by itself, be interpreted as evidence of neglect. Assessment of possible neglect should take into account the circumstances of the exposure, caregiver supervision and behavior, accessibility and storage of the substance, environmental hazards, and the overall context of the event. Deaths brought on by abuse or neglect, intentional administration of toxic chemicals, or caregivers’ failure to prevent dangerous exposures (Farrell et al., 2023). Another separate category is iatrogenic toxicity, which results from medical mistakes such improper drug delivery or dosage. Over time, long-term exposure to environmental factors like pesticides or heavy metals might have lethal consequences. Accurate forensic interpretation and preventative measures depend on these categories being properly classified (Grabska et al., 2022). As children grew older, the incidence of unintentional poisoning declined. When it came to unintentional poisoning, metropolitan youngsters were more likely to be male. Males and females were equally likely to become accidently poisoned in rural settings. According to reports, household products are the most frequent source of unintentional exposure. Rural children under 5 years old were more likely to be poisoned by common home items such pesticides, fuels, oils, solvents, and caustic compounds (Vilaca et al., 2020). Differentiating between the manner of death and the cause of death is crucial in forensic medicine. While the manner of death describes the medico-legal circumstances under which the fatal poisoning occurred (natural, accident, suicide, homicide, or undetermined), the cause of death refers to the specific pathological or toxicological process responsible for death (e.g., carbon monoxide poisoning or opioid intoxication). Table 2 summarizes the main medicolegal situations of pediatric poisoning deaths, along with their usual toxicological mechanisms, conditions, toxic substances, and important forensic signs.

### 5.1 Overview of manner of death classification

In forensic investigations of pediatric poisoning deaths, manner of death is commonly classified as accidental, suicidal, homicidal, or undetermined, based on toxicological findings, autopsy results, scene investigation, and clinical history. Differentiating between these categories can be challenging in children due to limited exposure history and dependence on caregiver information. Accidental poisonings predominate in younger children, while intentional exposures are more frequently observed in adolescents. Accurate classification is essential for medico-legal interpretation of pediatric poisoning cases (Krzyzaniak et al., 2016; Hannibal et al., 2025).

### 5.2 Natural vs. unnatural causes

In pediatric forensic toxicology, deaths are generally classified as natural or unnatural based on whether they result from disease processes or external factors such as poisoning or injury. Unnatural deaths, including poisoning-related fatalities, require careful investigation to distinguish between accidental, suicidal, and homicidal exposures (Gokhul et al., 2016). This differentiation is often challenging in children due to limited exposure history and reliance on caregiver information. Accurate classification is essential for medico-legal interpretation and identification of potentially preventable deaths (Wroblewski et al., 2025).

### 5.3 Role of toxicology in determination

Toxicology plays a central role in pediatric forensic investigations by enabling the detection, identification, and quantification of drugs, chemicals, and environmental toxins in biological samples, thereby contributing to the determination of cause and manner of death. However, interpretation of toxicological findings in children is complex due to age-dependent physiological differences, the absence of standardized pediatric reference data, and postmortem changes such as redistribution and decomposition, which may significantly alter drug concentrations. In addition, microbial activity, sample type, storage conditions, and postmortem interval can further influence analytical results. Therefore, toxicological findings should always be interpreted in conjunction with autopsy findings, scene investigation, clinical history, and circumstantial evidence to ensure accurate medico-legal conclusions (Charkiewicz et al., 2025).

A number of intrinsic limitations must be considered when interpreting postmortem toxicological results. The detection or concentration of a toxic substance in postmortem specimens should not be interpreted in isolation or considered, by itself, definitive proof of the cause of death. Toxicological findings must be interpreted in conjunction with the clinical history, circumstances of death, autopsy findings, specimen type, postmortem interval, potential postmortem redistribution, decomposition, storage conditions, and relevant toxicological and pharmacological information.

Drug concentrations after death may be considerably changed by postmortem redistribution, especially for lipophilic substances with a high volume of distribution. While microbial activity may produce postmortem ethanol, which could lead to an incorrect diagnosis of alcohol intoxication, decomposition can alter the stability of endogenous chemicals and xenobiotics. Analytical results may also be impacted by the specimen type, storage conditions, environmental factors, and postmortem interval (PMI). As a result, toxicological results should never be understood alone; rather, they should always be evaluated in conjunction with the results of autopsies, scene investigations, clinical histories, and circumstantial evidence (Twomey et al., 2013; Roman et al., 2014).

### 5.4 Postmortem specimen collection, preservation and chain of custody

Postmortem toxicological analysis in suspected pediatric poisoning requires standardized and prioritized collection of biological specimens to ensure diagnostic reliability. Peripheral (femoral) blood is the preferred matrix for quantitative analysis due to lower susceptibility to postmortem redistribution (Burton et al., 2014). When unavailable, alternative samples such as cardiac blood, urine, vitreous humour, and gastric contents should be collected. Solid tissues, including liver, kidney, and lung, may provide complementary information, particularly in decomposed cases or when conventional samples are limited (Shapiro-Mendoza et al., 2014).

Bile may be used as an additional auxiliary matrix when standard specimens are unavailable; however, its interpretation is limited due to lack of established reference values and postmortem redistribution effects, and it should be considered supportive rather than confirmatory. All specimens should be collected under standardized forensic protocols with proper documentation, labeling, and secure storage to preserve integrity. Chain of custody must be maintained throughout all stages of handling and analysis (Shapiro-Mendoza et al., 2014). Toxicological investigation should follow a two-step approach, consisting of initial immunoassay screening and confirmatory analysis using chromatographic techniques, preferably LC–MS/MS. Interpretation of results must be integrated with autopsy findings and scene investigation data to ensure accurate medico-legal conclusions (Tunesi et al., 2023).

## 6 Abuse-related poisoning intentional administration of toxins

According to the World Health Organization (WHO), child maltreatment encompasses all forms of physical and emotional ill-treatment, sexual abuse, neglect or negligent treatment, exploitation, and other acts resulting in actual or potential harm to a child’s health, survival, development, or dignity (Tunesi et al., 2023). Within this framework, pediatric poisoning represents an underrecognized manifestation of child maltreatment. Maltreatment-related poisoning may occur through intentional administration of toxic substances or medications (abuse) or through inadequate supervision, unsafe storage of hazardous substances, or failure to provide a safe environment (neglect). Although these mechanisms differ in intent, both are preventable and may result in severe intoxication or fatal outcomes. From a forensic perspective, maltreatment-related poisoning remains particularly challenging because affected children often present with non-specific manifestations that mimic accidental poisoning or natural disease. Accurate recognition therefore requires integration of clinical information, scene investigation, toxicological findings, post-mortem examination, and child protection assessment within a multidisciplinary framework (Woudenberg–van den Broek et al., 2022). The principal forensic characteristics of abuse- and neglect-related poisoning are summarized in Table 3.

### 6.1 Drug-facilitated child abuse

Drug-facilitated child abuse refers to the covert and intentional administration of pharmacological agents to a child without medical indication, most commonly for the purpose of sedation, control, or harm (Baljak et al., 2025). While it may overlap with Munchausen syndrome by proxy, it is distinguished by direct toxic exposure rather than the fabrication of illness for psychological gain. Reported agents include benzodiazepines, antihistamines, opioids, sedative-hypnotics, and anticoagulants, among others (Flaherty et al., 2013). The clinical recognition of such exposures is often complicated by the non-specific nature of symptoms and the absence of a clear exposure history, which may delay consideration of an exogenous cause. Toxicological analysis therefore represents a central component in the diagnostic process, although routine screening may fail to detect all relevant compounds, particularly in cases involving low-dose or intermittent exposure, necessitating targeted analytical approaches and careful interpretation of biological samples (Hines, 2016). In these cases, interpretation depends on the integration of toxicological findings with clinical and investigative context in order to distinguish between accidental ingestion, therapeutic error, and intentional exposure. Recurrent unexplained episodes, inconsistent clinical courses, or improvement following separation from the caregiver may further support suspicion of abuse. Post-mortem investigations, supported by toxicological, microbiological, and pathological findings, contribute to reconstructing the sequence of events and determining the cause and manner of death (Wood et al., 2018).

### 6.2 Case indicators and red flags

In cases of unexplained or suspicious pediatric death, particularly where abuse-related poisoning or Factitious Disorder Imposed on Another is considered, recognition of potential red flags remains challenging due to the non-specific and heterogeneous nature of findings. Suspicion is therefore based on the convergence of multiple inconsistencies across medical history, scene investigation, and post-mortem results rather than on a single defining feature. Contextual indicators may include the absence of a plausible natural disease explanation despite extensive evaluation, discrepancies between reported history and objective findings, and lack of findings consistent with the alleged clinical course. Recurrent unexplained fatal events within the same household further strengthen suspicion of non-accidental etiology, particularly when previous records reveal similar unresolved presentations (Oppan et al., 2024). Post-mortem findings may provide additional indirect indicators, including unexpected toxicological results, detection of xenobiotics not compatible with prescribed therapy, or biochemical inconsistencies across biological matrices. In suspected poisoning, discordant drug concentrations or abnormal electrolyte profiles may suggest exogenous administration, although interpretation may be limited by post-mortem redistribution and incomplete screening panels (Aly et al., 2020). Structural findings may also contribute, particularly in medically managed children, where evidence of tampering or inappropriate manipulation of devices such as feeding tubes, catheters, or shunts may raise further concern when considered alongside toxicological or pathological abnormalities. Overall, red flags in suspected abuse-related pediatric death are best interpreted as a pattern-based construct requiring integration of autopsy findings, toxicological data, and investigative context within a multidisciplinary framework. The absence of specific diagnostic markers remains a key limitation, underscoring the importance of maintaining a high index of suspicion in cases with unexplained or inconsistent findings.

### 6.3 Forensic autopsy protocol in suspected abuse-related poisoning

The forensic investigation of suspected abuse-related poisoning requires a systematic and multidisciplinary approach integrating the circumstances of death, scene investigation, complete autopsy examination, toxicological analyses, histopathology, and, where indicated, complementary laboratory investigations. Because many toxic exposures produce non-specific or even absent postmortem findings, neither autopsy nor toxicological analysis should be interpreted in isolation. Rather, determination of the cause and manner of death requires correlation of analytical findings with the medical history, circumstances of death, scene findings, and pathological examination. The investigation should include a comprehensive external examination and careful documentation of findings before internal examination. Particular attention should be paid to findings that may indicate toxic exposure or routes of administration, including puncture or injection marks, unusual livor or skin discoloration, material or froth around the mouth and nose, chemical injury, and other external abnormalities. In suspected abuse-related cases, these findings should be interpreted alongside the circumstances of death, available medical information, and evidence obtained during scene investigation, as individual external signs are rarely diagnostic of poisoning on their own (Pragst, 2025).

Whenever available and appropriate, postmortem imaging may be performed before conventional autopsy as an adjunct to the forensic examination. Postmortem computed tomography (PMCT) is particularly valuable for demonstrating skeletal and traumatic injuries, whereas postmortem magnetic resonance imaging (PMMRI) provides superior soft-tissue and intracranial detail. In suspected child abuse, these techniques may reveal occult injuries that are not immediately apparent at conventional examination and can assist in directing subsequent autopsy; however, imaging findings should be correlated with autopsy and histopathological findings rather than interpreted independently (Edwards et al., 2023; Kelly et al., 2026).

The internal examination should include systematic assessment of the major organ systems and careful inspection for pathological changes that may support or provide an alternative explanation for death. Particular attention should be given to the gastrointestinal tract and its contents in suspected poisoning, as tablets, unusual material, mucosal injury, or other findings may provide important clues regarding toxic exposure. Examination of the lungs, liver, kidneys, brain, and other organs may reveal findings compatible with toxic injury, although many such changes are non-specific. Consequently, gross pathological findings should be interpreted together with histopathological and toxicological results and the broader investigative context (Pragst, 2025).

Histological examination remains an important component of the investigation, as it may demonstrate microscopic evidence of toxic injury and help exclude natural diseases that can mimic poisoning. Findings such as hepatic injury, acute tubular damage, pulmonary edema or aspiration, and hypoxic changes should be interpreted together with toxicological and other postmortem findings rather than in isolation (Pragst, 2025).

Comprehensive toxicological analysis is central to the investigation of suspected poisoning. Whenever possible, specimens should include peripheral blood, urine, vitreous humour, gastric contents, and selected tissues such as liver, with additional matrices collected according to the circumstances of the case. Peripheral blood is generally preferred for quantitative interpretation because it is less affected by postmortem redistribution, while vitreous humour provides a relatively protected complementary matrix. Hair analysis may additionally assist in evaluating chronic or repeated exposure. Toxicological screening should be followed by appropriate confirmatory analysis using specific chromatographic–mass spectrometric techniques such as GC–MS or LC–MS/MS (Bévalot et al., 2016; Drummer, 2024; Pragst, 2025).

Microbiological and biochemical investigations should be considered when infection, metabolic disturbances, or other natural causes remain within the differential diagnosis. Postmortem biochemical analysis, particularly of vitreous humour, may provide additional information regarding electrolyte and metabolic abnormalities. In otherwise unexplained pediatric deaths, molecular investigations may also be considered when an inherited cardiac or metabolic disorder is suspected.

Ultimately, determination of the cause and manner of death requires integration of the circumstances of death, medical history, autopsy and imaging findings, histology, toxicology, and complementary laboratory investigations. A multidisciplinary approach is particularly important in suspected abuse-related poisoning, where analytical findings must be interpreted within the complete clinical and medicolegal context (Pragst, 2025; Drummer, 2024).

Forensic examination of child poisoning deaths using an integrated multidisciplinary strategy. Following a multidisciplinary case review and determination of the cause and manner of death, the framework integrates a scene investigation and case history with post-mortem imaging, autopsy and histopathology, toxicological analysis, post-mortem biochemistry and microbiology, and neuropathological assessment. Adapted and modified from Chatzinikolaou et al. (2025) under the Creative Commons Attribution (CC BY) license.

The original framework was modified by replacing the biomechanical modeling component with post-mortem biochemistry and microbiology to reflect the scope of the present review.

## 7 Neglect and toxic exposure

In pediatric populations, exposure to environmental toxins is closely linked to the quality and safety of the living environment, and often reflects prolonged absence of adequate protection rather than isolated events. Substances such as carbon monoxide, lead, and arsenic are most frequently encountered in domestic or community settings where hazards are persistent and largely preventable, arising from unsafe housing conditions, inadequate supervision, or environmental contamination. From a pathophysiological perspective, it is important to distinguish between acute, high-intensity exposures and chronic, low-level exposure patterns, as they differ in both clinical course and biological impact. Acute exposures are typically associated with rapid systemic deterioration, while chronic exposures lead to gradual accumulation of toxic burden and long-term organ dysfunction, particularly affecting the developing nervous system (Kozer et al., 2006). A structured overview of the main toxic agents (carbon monoxide, lead, and arsenic), together with their typical exposure patterns, underlying mechanisms of toxicity, and key clinical and post-mortem findings associated with both acute and chronic exposure, is presented in Table 3 below.

### 7.1 Environmental toxins (e.g., lead, carbon monoxide)

Exposure to toxic environmental agents in childhood should be considered not only within the framework of accidental poisoning, but also in the broader context of child neglect and failure to provide safe living conditions. In many forensic cases, such exposures reflect underlying environmental insecurity, inadequate supervision, or prolonged residence in hazardous housing conditions, rather than isolated toxicological events. Carbon monoxide exposure most commonly occurs in poorly ventilated domestic environments and is frequently linked to defective heating systems or unsafe household infrastructure. In pediatric fatalities, these circumstances often indicate preventable environmental neglect, where prolonged exposure risk was not identified or mitigated despite ongoing vulnerability (Karrari et al., 2012). Similarly, long-term shortcomings in environmental safety and child supervision are reflected in lead exposure, which is usually linked to deteriorating housing conditions, contaminated household dust, and other environmental risks. Arsenic-related deaths, although less frequent, are likewise often connected to environmental contamination or unsafe exposure sources, requiring careful consideration of living conditions in the forensic assessment (Tuakuila et al., 2013).

Inadequate ventilation, dangerous heating methods, poor housing conditions, and a lack of monitoring all contribute to circumstances where toxic exposures become predictable and avoidable. These exposures are rarely isolated incidents; rather, they are a reflection of ongoing inadequacies in identifying and mitigating environmental risks to children who are at risk. One significant but often overlooked route of hazardous exposure for children is through unsafe home environments. Defective heating systems, poorly ventilated areas, and inadequate fuels considerably increase the risk of carbon monoxide poisoning (Tuakuila et al., 2013).

### 7.2 Failure to provide safe living conditions

Inadequate living conditions may expose children to multiple preventable environmental hazards, particularly when poor housing quality, insufficient ventilation, unsafe heating practices, and inadequate maintenance occur simultaneously. Such conditions may facilitate repeated or prolonged exposure to toxic agents within the home environment. Unsafe or inappropriate household heating represents an important source of carbon monoxide exposure, particularly during colder months, while inadequate ventilation may further increase the risk of accumulation in enclosed spaces (Gozubuyuk et al., 2017). Deteriorating housing conditions may also increase children’s exposure to lead, particularly through deteriorated lead-based paint and contaminated household dust. Residential dust lead levels are influenced by housing age, paint deterioration, and renovation activities and are associated with increased blood lead concentrations in young children (Gaitens et al., 2009; Dixon et al., 2009). As a product of incomplete combustion of organic materials, carbon monoxide commonly arises in everyday domestic contexts, including the use of charcoal, gas, or petroleum-based fuels in poorly maintained or inadequately ventilated heating and cooking systems, as well as during house fires or exposure to exhaust emissions in enclosed areas (Karrari et al., 2012). Likewise, deteriorating housing infrastructure, particularly the presence of lead-based paint, contaminated dust, and environmental pollutants remains a well-established source of chronic toxic exposure. These risks are often amplified by inadequate hygiene, improper waste handling, and lack of safe water and food management, contributing to cumulative and overlapping hazards (Muruka et al., 2007; Schneider, 2023).

The impact of such environments is intensified by the inherent vulnerability of children, whose behavioral patterns and developmental physiology facilitate greater exposure and reduced capacity for detoxification. Prolonged contact with environmental toxins may therefore remain clinically unrecognized until advanced or fatal outcomes occur. Such conditions are particularly pronounced in socioeconomically disadvantaged settings, where environmental hazards, overcrowding, and limited access to safe infrastructure further increase the risk of sustained toxic exposure in children. In the medicolegal evaluation of these cases, interpretation extends beyond the identification of toxic agents alone. Reconstruction of the child’s living environment becomes essential, as the presence of preventable hazards particularly when persistent or unaddressed may indicate a broader pattern of neglect underlying the exposure (Khalequzzaman et al., 2011).

### 7.3 Chronic vs. acute exposure patterns

The manner in which toxic exposure contributes to pediatric fatalities is shaped by the intensity, duration, and context of contact with the toxic agent. In high-intensity, short-term exposures, clinical deterioration is typically rapid, following a single or substantial dose. Such scenarios are often associated with mechanisms like acute systemic hypoxia, as observed in carbon monoxide poisoning, or rapid multi-organ toxicity, as in acute arsenic exposure (Rastogi et al., 2019). The clinical course is usually brief, with progression to respiratory failure or cardiovascular collapse occurring within a limited time frame. Post-mortem findings in these cases are frequently non-specific, making toxicological analysis essential for confirming the cause of death (Di Sarno et al., 2025).

The substantial role of acute hazardous exposures to pediatric mortality is supported by epidemiological data. Poisoning is a major cause of accidental childhood fatalities, according to autopsy-based research, and carbon monoxide (CO) is often linked to improper heating techniques and poorly ventilated homes (Kinoshita et al., 2020). Inadequate housing, a reliance on solid fuels, and restricted access to clean water, sanitary facilities, and other essential services raise the risk of toxic exposure and related morbidity, all of which have an impact on exposure patterns (Rastogi et al., 2019; Kinoshita et al., 2020).

On the other hand, long-term exposure to environmental pollutants, notably lead, causes cumulative damage to multiple systems, with the developing nervous system being particularly susceptible. Persistent neurodevelopmental, cognitive, and behavioral impairments that continue throughout later life may result from even low-level exposure (Gözübüyük et al., 2017).

In severe situations, chronic toxic exposure can proceed to systemic decompensation affecting many organs and neurological degeneration, sometimes staying clinically undetected until advanced stages. Consequently, forensic examination needs not only toxicological confirmation but also investigation of long-term environmental exposure and living situations.

Importantly, acute and chronic toxic exposures usually result from the same dangerous environment. This overlap emphasizes that failing to provide safe living conditions represents a continuum of hazardous dangers, ranging from acute life-threatening incidents to chronic exposures with lasting developmental repercussion. Table 3 summarizes the main toxic substances linked to juvenile poisoning, along with their normal exposure patterns, mechanisms of toxicity, acute and chronic symptoms, and forensic importance.

## 8 Accidental poisoning in children

During early childhood, natural curiosity and exploratory behavior increase the risk of unintentional exposure to toxic substances, particularly medications and household products that are accessible within the home. Most exploratory ingestions produce no or only limited clinical effects; however, certain substances may cause severe toxicity or death even following relatively small exposures (O'Donnell, 2017; Berg and McCann, 2023).

Unsafe storage of medications and inadequate caregiver knowledge are important preventable risk factors for childhood poisoning, while delayed access to medical care has been associated with poorer outcomes and increased mortality (Mottla et al., 2023). Although fatal poisoning in young children is uncommon, deaths continue to occur, with medications—particularly opioids and over-the-counter preparations—among the substances implicated in reported fatalities (Gaither et al., 2024). These findings emphasize the importance of safe storage, caregiver education, early recognition, and timely medical intervention. The principal mechanisms, risk factors, toxic agents, and preventive approaches related to accidental childhood poisoning are summarized in Table 4.

**TABLE 4:** Overview of accidental poisoning in children.

Poisoning	Main risk factors	Common agents/issues	Key implications	References
Household chemicals and medications	Unsafe storage, low caregiver awareness, delayed recognition	Cleaning agents, corrosives, kerosene, pesticides, medications	Preventable exposure, forensic reconstruction	(Pragst, 2025; Keka et al., 2014; Heard, 2018)
Packaging and supervision	Improper closure, inadequate monitoring, unsafe placement	Failure of CRC use, observational learning, access through repetition	Neglect assessment, event reconstruction	(Karabacak et al., 2026; Heard, 2018; Mottla et al., 2023)
Paracetamol overdose	Dosing errors, multiple-product use, delayed treatment	NAPQI toxicity, hepatotoxicity, repeated supratherapeutic exposure	Liver injury evaluation, medico-legal causality	(Bouvet et al., 2020; Ahmed et al., 2022)
Prevention strategies	Insufficient education, weak regulation, delayed response	Poor labelling, lack of counselling, limited poison-centre awareness	Reduced morbidity, improved public health surveillance	(Keka et al., 2014; Mottla et al., 2023

### 8.1 Household chemicals and medications

In many cases, neglect-related toxic exposure is closely linked to accidental poisoning by household chemicals and medications, particularly in younger children whose developmental curiosity and limited risk awareness make them especially vulnerable. Most exposures occur in the home, usually involving substances left within reach or stored improperly. Children between one and 5 years of age are most frequently affected, which reflects their exploratory behaviour and inability to recognize danger (Keka et al., 2014). Although most pediatric poisonings result in mild outcomes, fatal cases continue to be reported. A retrospective study recorded 46 deaths among 8,802 acute poisoning cases (0.52%), with caustic agents as the leading cause, followed by carbon monoxide, pharmaceuticals, and pesticides. These findings are forensically important because such deaths often involve preventable environmental circumstances and may indicate inadequate supervision or unsafe storage practices (Singh et al., 2023). Alkali-based drain and oven cleaners are particularly dangerous due to their ability to cause deep tissue necrosis and delayed airway compromise, while substances such as hydrofluoric acid may cause rapid systemic toxicity with minimal visible external injury, complicating postmortem interpretation. Pharmaceutical poisoning is also frequently encountered, especially with analgesics, sedatives, and cardiovascular medications that are easily accessible to children (Singh et al., 2023). In fatal corrosive exposures, postmortem examination commonly demonstrates severe mucosal burns, airway edema, and respiratory collapse. From a forensic perspective, interpretation requires correlation of toxicological findings with scene investigation, autopsy findings, and developmental context. Easily accessible toxins, inappropriate packaging, delayed emergency response, or inconsistent caregiver accounts may support concerns of neglect rather than purely accidental exposure. Beyond establishing cause of death, forensic toxicology therefore plays an important role in identifying preventable environmental risk factors and informing future poison prevention strategies (White et al., 2018).

While some compounds, like hydrofluoric acid, can induce life-threatening systemic poisoning despite little outward symptoms, corrosive substances may cause significant tissue damage and delayed airway impairment. Analgesics, sedatives, and cardiovascular medications are the most frequently involved in pharmaceutical poisonings (Karabacak et al., 2026).

As a result, forensic toxicology helps identify avoidable environmental risk factors and support future poisoning prevention measures in addition to helping determine the cause of death (White et al., 2018).

### 8.2 Role of packaging and supervision

Evidence consistently shows that the risk of unintentional pediatric poisoning is strongly influenced by two interconnected factors: the design of packaging and the level of caregiver supervision. Although child-resistant containers (CRCs) and regulatory measures have significantly reduced poisoning incidence over past decades, they do not eliminate risk, particularly when supervision is inadequate or storage practices are unsafe (Dayasiri et al., 2017).

Child-resistant packaging has been one of the most effective public health interventions in reducing pediatric poisoning. Large population studies have demonstrated marked declines in unintentional ingestion and mortality following its introduction, including reductions of nearly 45% in fatal medication-related poisonings in children under five. Similarly, long-term surveillance data indicate sustained decreases in hospital admissions and poisoning-related deaths after mandatory implementation of safety packaging regulations. However, CRCs function primarily as a delaying mechanism rather than a complete barrier, giving time for adult intervention rather than fully preventing access (Morrongiello et al., 2024). Despite these benefits, studies show that more than half of pediatric poisoning cases still involve substances stored in compliant child-resistant packaging. This highlights a critical limitation: packaging effectiveness depends heavily on correct use and storage. Experimental research further demonstrates that children, particularly those aged five to 8 years, may successfully open CRCs after observing adult demonstration, indicating that modelling behaviour significantly increases access risk. In addition, repeated exposure opportunities and unsafe storage practices further enhance the likelihood of successful access to toxic substances. Accordingly, child-resistant packaging should be implemented for all over-the-counter medications, as these products are frequently present in households and represent a common source of accidental pediatric exposure (Karabacak et al., 2026; Heard, 2018). Supervision plays an equally important role but is often overestimated by caregivers. Research shows that parental perception of adequate supervision does not always align with actual monitoring behaviours, and even short periods of reduced attention can allow exposure. Developmental studies indicate that lower impulse control in children is associated with increased interaction with hazardous substances, while older children may also be at risk due to reduced parental vigilance as independence increases. From a forensic perspective, packaging and supervision are central to reconstructing poisoning events.

### 8.3 Common agents

Paracetamol (acetaminophen) is one of the most widely used analgesic and antipyretic agents in paediatric practice due to its well-established efficacy and safety at therapeutic doses. However, its extensive availability and inclusion in numerous over-the-counter preparations make it a frequent cause of both accidental and intentional poisoning, including severe and potentially fatal hepatotoxicity (Bouvet et al., 2020; Shekunov et al., 2021). In young children, acute toxicity most commonly results from unintentional overdose, often due to dosing errors by caregivers or the simultaneous use of multiple paracetamol-containing products. In adolescents, intentional ingestion, including suicidal attempts, becomes more relevant. Although paracetamol is generally safe when used appropriately, its narrow therapeutic margin means that relatively small deviations above recommended doses may result in clinically significant liver injury. The pathophysiology of toxicity is well understood. In overdose conditions, hepatic glucuronidation and sulfation pathways become saturated, leading to increased metabolism via the cytochrome P450 system and the formation of the reactive metabolite N-acetyl-p-benzoquinone imine (NAPQI). Under normal circumstances, NAPQI is detoxified by glutathione. However, when glutathione stores are depleted, NAPQI binds to hepatocellular proteins, triggering oxidative stress, mitochondrial dysfunction, centrilobular necrosis, and potentially acute liver failure (Ahmed et al., 2022). Clinical outcomes depend on the ingested dose, the timing of N-acetylcysteine administration, and individual susceptibility factors, including nutritional status, comorbidities, and possible genetic or metabolic predispositions. While severe outcomes are less common in children compared with adults, the literature documents cases of significant hepatotoxicity and even fatal outcomes, particularly in repeated supratherapeutic ingestion or delayed treatment. From a forensic and medico-legal perspective, paracetamol poisoning represents a complex diagnostic challenge. The assessment of causality relies on toxicological findings, clinical presentation, and histopathological features, most typically centrilobular hepatic necrosis, sometimes accompanied by microvesicular steatosis. Interpretation is often complicated by discrepancies between reported and actual ingested doses, as well as the potential contribution of underlying metabolic vulnerabilities (Heard, 2018).

### 8.4 Prevention strategies

Since most paediatric poisonings occur in the household, medications and toxic household chemicals should be stored in locked cabinets, kept in their original labelled containers, and placed out of children’s reach to reduce unintentional exposure (Keka et al., 2014). Additional regulatory measures, including improved warning labels and restricted package sizes for high-risk substances, may further reduce accidental exposure (Mottla et al., 2023). A multidisciplinary strategy combining caregivers, medical experts, poison control facilities, and public health authorities is necessary to prevent pediatric poisoning. The cornerstones of preventing unintentional poisoning continue to be the safe storage of pharmaceuticals and home chemicals, child-resistant packaging, caregiver education, and precise dosage recommendations. While legislative actions, public awareness campaigns, and surveillance systems can lower avoidable poisoning-related morbidity and mortality, prompt consultation with poison control centers and prompt medical intervention enhance clinical results. To improve child protection and preventive measures, special focus should be placed on identifying environmental risks and neglect-related risk factors (Kyle et al., 2026). The overview patterns of accidental poisoning in children, including associated risk factors, common toxic agents, key clinical and forensic implications, and relevant prevention strategies, are summarized in Table 4 below.

### 8.5 Iatrogenic toxicity and medical malpractice in paediatric poisoning deaths

Medication errors represent an important and preventable cause of harm in pediatric patients and may occur during prescribing, dispensing, preparation, or administration of medications. Children are particularly vulnerable because dosing frequently requires individual calculations based on body weight, age, or body surface area, while developmental differences in pharmacokinetics and pharmacodynamics further complicate drug therapy. Consequently, dosing errors are among the most frequently reported medication errors in pediatric practice and may result in clinically significant toxicity (Ghaleb et al., 2010; Conn et al., 2019; Koeck et al., 2021).

From a patient-safety perspective, pediatric medication errors should be considered within the broader medication-use system rather than solely as isolated individual failures. Contributing factors include dose-calculation errors, communication problems, inadequate pediatric formulations, insufficient decision support, and lack of standardized pediatric prescribing and dosing processes. Strategies such as computerized prescribing with pediatric-specific clinical decision support, standardized dosing protocols, and pharmacist involvement may reduce preventable errors (Conn et al., 2019; Koeck et al., 2021). In forensic pediatric toxicology, iatrogenic toxicity is an important differential consideration in cases of suspected poisoning fatalities. Determining whether a fatal outcome is related to medication error requires integration of clinical documentation, toxicological findings, and case circumstances to distinguish it from accidental or intentional exposure (Basilicata et al., 2024; Tzimenatos et al., 2009).

## 9 Conclusion

Paediatric toxicological deaths represent a complex and multifactorial medico-legal and public health issue, encompassing accidental poisoning, environmental exposure, neglect, and intentional administration of toxic substances. Children are uniquely vulnerable to toxic agents because of developmental physiological and pharmacokinetic differences that influence absorption, distribution, metabolism, and elimination of toxins. These factors contribute to increased susceptibility even at relatively low exposure levels and complicate both clinical management and forensic interpretation.

The role of forensic toxicology is essential in determining the cause and manner of death, particularly in cases involving covert poisoning, chronic exposure, or suspected child abuse. Recognition of clinical indicators, environmental risk factors, and inconsistencies in caregiver history remains critical for early identification and intervention.

Despite advances in toxicological analysis and preventive strategies, paediatric toxicological deaths continue to pose significant diagnostic and investigative challenges. Underreporting, variability in death classification, and limitations in postmortem toxicology may hinder accurate identification of poisoning-related fatalities. Therefore, multidisciplinary collaboration among clinicians, forensic specialists, toxicologists, social services, and public health professionals is essential to improve detection, investigation, prevention, and child protection outcomes.

Future research and clinical efforts should focus on improving the early recognition, investigation, and prevention of paediatric toxicological deaths through multidisciplinary and internationally standardized approaches.

One important priority is the development of standardized forensic and toxicological protocols for paediatric death investigation. Variability in autopsy practices, toxicological screening panels, specimen collection, and interpretation of postmortem findings may contribute to underdiagnosis or misclassification of toxicological deaths. International consensus guidelines specifically tailored to paediatric populations are needed to improve diagnostic consistency and medico-legal accuracy.

Another important direction involves strengthening surveillance and epidemiological reporting systems. Improved national and international poison surveillance databases could facilitate earlier identification of emerging toxic threats, regional exposure patterns, high-risk populations, and trends related to pharmaceutical agents, household chemicals, illicit substances, and environmental contaminants. Harmonized reporting systems would also improve comparability between studies and support evidence-based prevention strategies. Additional attention should be directed toward the forensic recognition of abuse-related and neglect-associated poisoning. Covert poisoning, fabricated illness, and intentional toxic exposure remain underrecognized forms of child maltreatment. Future studies should aim to establish validated clinical and forensic indicators that assist healthcare professionals, forensic experts, and child protection services in identifying suspicious cases at earlier stages. Technological advancements may also improve the field of paediatric forensic toxicology. Emerging analytical methods, including high-resolution mass spectrometry, metabolomics, toxicogenomics, and biomarker-based approaches, may increase the sensitivity and specificity of toxin detection, particularly in cases involving low-dose, delayed, or chronic exposure. Integration of artificial intelligence and predictive modelling into toxicological risk assessment and death investigation may further enhance diagnostic accuracy.

From a public health perspective, future preventive strategies should prioritize community education, safer medication storage, child-resistant packaging, environmental safety regulations, and targeted interventions for vulnerable families and high-risk households. Greater collaboration between healthcare systems, poison control centres, forensic institutions, schools, and social services is essential to reduce preventable paediatric toxic exposures and fatalities.

Ultimately, paediatric toxicological deaths require a comprehensive interdisciplinary framework that integrates clinical toxicology, forensic medicine, epidemiology, child protection, and public health policy. Continued research and international collaboration will be essential for improving prevention, detection, forensic investigation, and long-term child safety outcomes.

To further lower the number of pediatric poisoning deaths, future initiatives should concentrate on bolstering poison prevention programs, enhancing caregiver education, increasing access to poison control facilities, and encouraging interdisciplinary cooperation between medical professionals, forensic experts, and public health authorities.

## Supplementary material

SUPPLEMENTARY APPENDIX FIGURE SA Workflow-oriented summary of pediatric poisoning, integrating exposure sources and circumstances, toxicokinetic and toxicodynamic factors, clinical outcomes, epidemiological aspects, laboratory assessment, and prevention and control strategies. Created in BioRender. Ajanovic T. (2026) https://BioRender.com/9ubzcaz, licensed under Academic licences.
